# Dendritic Cells Primed with *Paracoccidioides brasiliensis* Peptide P10 Are Therapeutic in Immunosuppressed Mice with Paracoccidioidomycosis

**DOI:** 10.3389/fmicb.2017.01057

**Published:** 2017-06-14

**Authors:** Leandro B. R. Silva, Lucas S. Dias, Glauce M. G. Rittner, Julián E. Muñoz, Ana C. O. Souza, Joshua D. Nosanchuk, Luiz R. Travassos, Carlos P. Taborda

**Affiliations:** ^1^Laboratory of Medical Mycology, Tropical Medicine Institute USP-LIM53, University of São PauloSão Paulo, Brazil; ^2^Department of Microbiology, Institute of Biomedical Sciences, University of São PauloSão Paulo, Brazil; ^3^Department of Medicine, Albert Einstein College of Medicine, BronxNY, United States; ^4^Department of Microbiology and Immunology, Albert Einstein College of Medicine, BronxNY, United States; ^5^Department of Microbiology, Immunology and Parasitology, Federal University of São PauloSão Paulo, Brazil

**Keywords:** *Paracoccidioides brasiliensis*, paracoccidioidomycosis, P10, adjuvants, dendritic cells, vaccine

## Abstract

Paracoccidioidomycosis (PCM) is an endemic systemic mycosis in Latin America, with the highest prevalence in Brazil, Colombia, and Venezuela. Fungi of the *Paracoccidioides* genus are etiologic agents of the disease. The 15 amino acid peptide P10 is derived from gp43, the main diagnostic antigen of *Paracoccidioides brasiliensis.* We previously reported that P10-pulsed dendritic cells (DCs) induce a protective response against *P. brasiliensis*. Presently, dexamethasone-treated BALB/c mice were intratracheally infected with *P. brasiliensis* Pb18 to establish the therapeutic efficacy of P10-pulsed DCs. Immunosuppressed and infected animals that received DCs had a reduction in their fungal burden, and this result was most pronounced in mice receiving DCs primed with P10. The efficacy of therapeutic DCs was significantly augmented by concomitant treatment with trimethoprim-sulfamethoxazole. Additionally, primed-DCs with or without the antifungal drug induced a beneficial Th_1_-biased immune response and significantly reduced tissue damage. In conclusion, these studies with immunocompromised mice demonstrate that P10-pulsed DCs with or without concomitant antifungal drugs are potently effective in combating invasive PCM. These findings support further translational studies to validate the use of P10-primed DCs for PCM in immunocompetent and immunosuppressed hosts.

## Introduction

Paracoccidioidomycosis (PCM), caused by thermally dimorphic fungi of the *Paracoccidioides* genus, is one of the most important systemic granulomatous diseases in Latin America (Taborda et al., 2015). PCM is particularly prevalent in Brazil, affecting mainly rural workers (Restrepo, 1985; Travassos and Taborda, 2012b). In Brazil, approximately 1,853 (∼51.2%) of 3,583 confirmed deaths due to systemic mycoses from 1996 to 2006 were caused by PCM (Prado et al., 2009). Phylogenetic analyses have revealed that the clinically relevant *Paracoccidioides* species include *Paracoccidioides brasiliensis*, comprised of the three cryptic species S1, PS2, and PS3 (Theodoro et al., 2012), and *Paracoccidioides lutzii* (Teixeira et al., 2013).

Acquisition of *Paracoccidioides* spp. follows the inhalation of conidia, which are deposited into the lower respiratory tract. These propagules subsequently undergo morphogenic transformation into yeast forms, which constitute the pathogenic morphology in tissues (Taborda et al., 2015). PCM has two main clinical forms that are predicated upon the immunological status of the infected host. Within weeks to months after initial infection, young adults often develop the acute or subacute forms of disease, and these are typically aggressive and require immediate antifungal treatment (Bocca et al., 2013). The chronic form of PCM is manifested months or years after infection, and disease varies in severity, although this form can be as aggressive as the acute form (Bocca et al., 2013).

Although polyene and azoles as well as the combination of trimethoprim and sulfamethoxazole are the typical therapeutics administered to patients with PCM, antifungal treatment is prolonged, frequently over 2 years, and failures and/or relapses occur (Travassos et al., 2008b; Bocca et al., 2013).

An experimental vaccine against *P. brasiliensis* has been studied (Travassos and Taborda, 2012b) using a 15 mer peptide, known as peptide 10 or P10, with the sequence: QTLIAIHTLAIRYAN (Taborda et al., 1998). P10 is derived from the major diagnostic antigen of PCM, the 43,000 Daltons glycoprotein known as gp43 (Travassos et al., 1995). Over the past 10 years, the properties and several different forms of delivery of P10 have been studied, and have contributed to validating this peptide as a potential candidate for a human vaccine (Iwai et al., 2003; Travassos et al., 2008a; Travassos and Taborda, 2012b; Taborda et al., 2015). Using dexamethasone-treated mice, immunization with P10 has been shown to efficiently modulate the immune response in immunosuppressed hosts. Protection against pulmonary challenge with *P. brasiliensis* has been demonstrated by increased animal survival, reduced lung fungal burden and reduced pulmonary fibrosis in P10 immunized mice as compared to control animals (Muñoz et al., 2014).

We previously demonstrated protective immunity against murine *Paracoccidioides brasiliensis* infection after sub-cutaneous or intravenous injection of P10-primed dendritic cells (DCs). Mice receiving the P10-primed DCs had a mixed cytokine response pattern with a predominance of Th_1_-type activation and a significant reduction of fungal burdens in comparison with control animals. This data supported our proposal that P10-primed DCs are promising therapeutics in the setting of established fungal infection (Magalhães et al., 2012; Thind et al., 2015). DCs are powerful inducers of T-lymphocyte immune responses against *Paracoccidioides* antigens (Magalhães et al., 2012; Thind et al., 2015). The administration of *in vitro* gp43-pulsed DCs into mice results in increased productions of IL-2 and IFN-γ by CD4^+^ T cells isolated from regional lymph nodes (Ferreira et al., 2003). There is significant interest in adoptive transfer of DCs as a means to harness T-cell mediated immunity as a therapy to combat pathogenic fungi, which is demonstrated by studies with DCs pulsed with fungal cells or with fungal RNA from *Candida albicans*, *Aspergillus fumigatus*, *Cryptococcus gattii*, and *Cryptococcus neoformans* (D’Ostiani et al., 2000; Bozza et al., 2003; Siegemund and Alber, 2008; Roy and Klein, 2012; Ueno et al., 2015, 2016).

However, there is yet limited information as to whether this modality of cell-induced therapy would be effective in the setting of immunocompromised patients, which is important since PCM is a frequent mycological cause of morbidity and mortality in patients with HIV in Brazil (Prado et al., 2009). In the present work, we simulated the acute/subacute form of PCM in immunocompromised mice by administering dexamethasone, which suppresses host immune responses through multiple mechanisms, including modulating inflammatory cytokines (Chung et al., 2011; Muñoz et al., 2014) prior to infection. Our findings show that the administration of P10-primed DCs to dexamethasone suppressed mice infected with *P. brasiliensis* are therapeutic in the setting of compromised immunity.

## Materials and Methods

### Animal Use and Ethics Statement

BALB/c mice (6-to 8-week-old, males) used for infection and bone marrow harvesting were bred at the School of Medicine – University of São Paulo in pathogen-free conditions. All animal experiments were performed in strict accordance with the Brazilian Federal Law 11,794 establishing the Animal Protection Code for the State of São Paulo. This study was approved by the Ethics Committee on Animal Experiments at the School of Medicine, University of São Paulo (189/14).

### Fungus and Inoculum Preparation

The virulent isolate *P. brasiliensis* Pb18 was cultivated on solid Sabouraud medium at 37°C for 7 days. The fungus was collected and washed three times in phosphate buffered saline (PBS, pH 7.2). After decanting large and agglutinated cells, small isolated yeast cells were counted in a Neubauer chamber. The yeast cells used for experimentation displayed >95% viability as determined by staining with Trypan blue.

### Immunosuppressed Mice

The animals were immunosuppressed as previously described (Muñoz et al., 2014). Dexamethasone phosphate (Sigma, St Louis, MO, United States) was administrated daily in drinking water and the dose was calculated as 0.15 mg/kg considering an average water intake of 5 ml per day. Dexamethasone phosphate treatment was initiated 20 days before intratracheal (i.t.) infection and continued until the end of experiment. Immunosuppression was confirmed by the blood leukocyte levels in comparison with untreated mice. Animals were maintained in isolator cages. Cages were exchanged twice a week in a laminar flow hood and all bedding, food and water was autoclaved prior to use. Total leukocyte counts were determined using ∼200 μl of blood obtained via the ocular plexus. The blood was mixed with 20 μL of ACD (citric acid, sodium citrate, dextrose) to prevent clot formation and then Turk solution was used to hemolyze erythrocytes and stain leukocytes. Leukocyte counting was performed using blood smears on glass slides. Blood samples were also counted in a Neubauer chamber.

### Intratracheal Infection

BALB/c mice were infected intratracheally (i.t.) with *P. brasiliensis* Pb18 20 days after immunosuppression was initiated. For the procedure, animals were anesthetized intraperitoneally using ∼200 μl of a solution containing 80 mg/kg ketamine and 10 mg/kg xylazine (both from União Química Farmacêutica, Brazil). Five minutes after the injection of the anesthetics, a small longitudinal skin incision was made in the neck to expose the trachea and 3 × 10^5^ yeast cells in 50 μl of PBS was injected. The incision was sutured with 5-0 silk.

### Peptide Synthesis and Purification

P10 peptide with amidated C-terminal was purchased from Peptide 2.0 (Chantilly, VA, United States). HPLC and MS analyses performed by the manufacturer showed that the synthetic P10 was 98% pure.

### Dendritic Cells Differentiation

Dendritic cells were obtained from bone marrow as described previously (Inaba et al., 1992). Briefly, femurs and tibias were collected from male BALB/c mice and flushed with RPMI (Vitrocell, Campinas, Brazil). Samples containing 10^7^ cells were cultivated in RPMI supplemented with 10% fetal calf serum (FCS; Vitrocell), 20 μg/ml gentamicin (Gibco BRL Life Technologies, Grand Island, NY, United States) and recombinant cytokines GM-CSF (30 ng/ml) and IL-4 (15 ng/ml) (both from Peprotech, Rocky Hill, NJ, United States) in 25 cm^2^ cell culture flasks. On the second day, 80% of medium was removed and the same amount of fresh medium with growth factors was added. On the fourth, sixth, and eighth day, cell supernatants were centrifuged and pellets were plated in fresh medium containing growth factor. On the ninth day, cells were harvested and used in the experiments.

### Expression of Costimulatory Molecules and Kinetics of Cytokine Production on DCs Pulsed with P10 or IFN-γ

Dendritic cells were distributed into 24-well plates at a 10^6^/ml cell density and cultivated in medium alone or with P10 (2.55 × 10^-1^mM) at 5% CO_2_ and 37°C. After 2 h, cells were removed, washed in FACS buffer and incubated with antibodies to CD11c (BV711, Clone N418), MHC-II (APC-Cy7, Clone M5/114.15.2), CD86 (APC, Clone 16-10A1) and CD-80 (PE, GL1) for 30 min at 4°C. Cells were analyzed by flow cytometry (BD FACSCanto) to assess the expression of surface molecules. In addition, culture supernatants of DCs pulsed with or without P10 or IFN-γ (20 ng/ml) for 2, 12, and 24 h were assessed for IL-10 and IL-12 production using enzyme-linked immunosorbent assay (ELISA) kits (BD OpTeia, San Diego, CA, United States).

### *In Vitro* Activation of DCs and Treatment of Infected Mice

Dendritic cells (10^6^/ml) were distributed into 24-well plates and cultivated in medium alone or with P10. After 2 h, cells were harvested and a total of 3 × 10^5^ cells were injected subcutaneously in immunosuppressed mice 30 days post-infection. Mice were also randomized to receive either trimethoprim-sulfamethoxazole (TMP-SMX) 3–15 mg/kg every day for 15 days. Controls groups were treated with PBS.

### Fungal Burden in Organs of Infected Mice

Mice were euthanized 45 days after infection (day 65 of experiment) and lungs were removed. Portions of lung were weighed, homogenized in PBS and aliquots were plated on agar brain heart infusion (BHI) supplemented with 4% fetal bovine serum (Gibco, Grand Island, NY, United States), 5% supernatant of spent culture of *P. brasiliensis* Pb192, 10 IU/ml streptomycin-penicillin (Cultilab, Brazil) and 500 mg/ml cycloheximide (Sigma, St. Louis, MO, United States) (Moscardi-Bacchi et al., 1994). Plates were incubated at 37°C for 20 days, and the resulting Colony Forming Units (CFUs) were enumerated.

### Cytokine Detection

Lung sections were homogenized in PBS with protease inhibitors: benzamidine HCl (4 mM), EDTA disodium salt (1 mM), *N*-ethylmaleimide (1 mM), and pepstatin (1.5 mM) (Sigma, St. Louis, MO, United States). Supernatants were assayed for IL-4, IL-10, IL-12, and IFN-γ using ELISA kits (BD OpTeia, San Diego, CA, United States). The detection limits of the assays were as follow: 7.8 pg/ml for IL-4, 31.3 pg/ml for IFN-γ and IL-10, and 62.5 pg/ml for IL-12, as determined by the manufacturer.

### Histological Analyses

Additional lung sections were fixed in 10% buffered formalin and embedded in paraffin for cutting. Sections were stained by the Gomori-Grocott method and hematoxylin/eosin (HE).

### Statistical Analysis

Data shown are representative of at least two independent experiments. For *in vivo* experiments, a total of six animals per group were used. *In vitro* experiments were done in triplicates for each condition. Statistical analyses were performed using GraphPad Prism 5 software (San Diego, CA, United States). The results were expressed as mean values and standard deviations (SD) of the indicated values. Tukey’s significant difference test was employed for non-parametric data. *p*-values of ≤0.05 were used to indicate statistical significance.

## Results

### *In Vitro* Characterization of DCs

**FIGURE 1:**
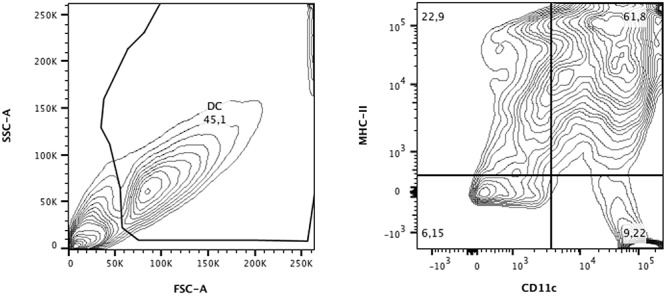
Flow cytometric analysis of dendritic cells (DCs) from BALB/c differentiated for 9 days in medium containing GM-CSF and IL-4. Cells were separated according to granularity (SSC) and size (FSC) and double-labeling for CD11c and MHC-II was evaluated. In CD11c^+^/MHC-II^+^ populations (∼60%), expression of CD80 and CD86 was also defined. Data were analyzed using FlowJo software (Tree Stars Inc.).

Dendritic cells from BALB/c mice were analyzed by flow cytometry. Cell were first gated by granularity (SSC) and size (FSC) and then analyzed for CD11c and MHC-II expression. Double positive cells for CD11c and MHC-II were considered as differentiated DCs, reaching a frequency of appropriately 60% (**Figure 1**), as previously observed (Magalhães et al., 2012). Therefore, for *in vivo* experiments, each vaccine dose contained a total of 3 × 10^5^ cells, of which 1.8 × 10^5^ were DCs pulsed or not with P10.

### Activation Dendritic Cells by Peptide P10

**FIGURE 2:**
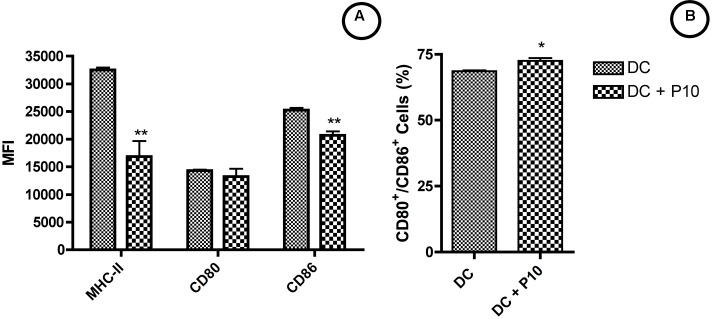
Activation of DCs by peptide P10. DCs were analyzed for MHC-II, CD80 and CD86 expression **(A)** and frequency of CD80^+^/CD86^+^ cells **(B)**. Analysis by one-way ANOVA followed by Tukey’s post-test, where ^∗^*p* < 0.05, ^∗∗^*p* < 0.01 in comparison with unstimulated group (DC).

We assessed the expression of MHC-II, CD80, and CD86 molecules within MHCII^+^/CD11c^+^ cell population after 2 h of stimulation with or without P10. According to the Median Fluorescence Intensity (MFI), we observed a decrease in MHC-II and increase of CD86 expression for P10-pulsed DCs and no significant changes for CD80 in comparison to control group (**Figure 2A**). However, the percentage of CD80^+^/CD86^+^ DCs showed a significant increase (**Figure 2B**), demonstrating that P10 might have a stimulatory effect for DCs activation.

### Measurements of IL-10 and IL-12 Cytokines in Culture Supernatants of *Ex Vivo* P10-Pulsed Dendritic Cells

**FIGURE 3:**
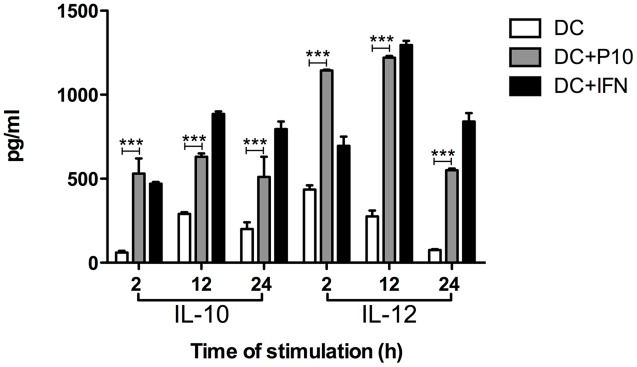
Measurement of IL-10 and IL-12 cytokines in culture supernatants of differentiated DCs *ex vivo* pulsed or not with P10 or IFN-γ. Supernatants of DCs incubated in medium alone (DC; negative control) or with either P10 (DC + P10) or IFN-γ (DC + IFN; positive control) were tested for IL-10 and IL-12 production. Analysis by one-way ANOVA followed by Tukey’s post-test, yielding ^∗∗∗^*p* < 0.001.

Dendritic cells pulsed with P10 showed increased production of IL-10 and IL-12 compared with untreated DCs (**Figure 3**). P10 induced an increased production of these cytokines, which was even higher in DCs pulsed with IFN-γ, although these levels were higher in the setting of IFN-γ at 12 and 24 h.

### Immunosuppression of BALB/c Mice with Dexamethasone

**FIGURE 4:**
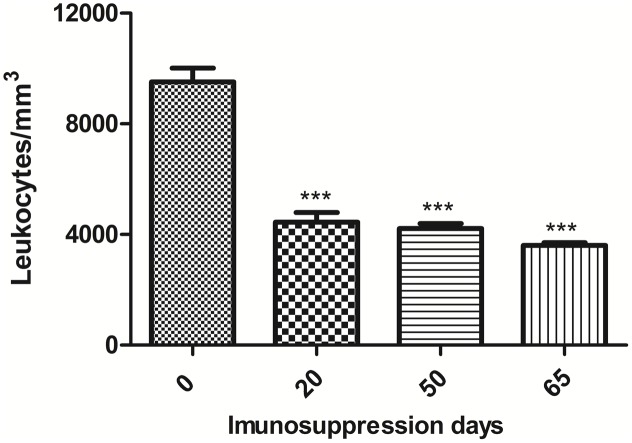
Total leukocyte counts in peripheral blood of BALB/c mice treated with dexamethasone. Leukocyte counts of mice prior to the initiation of daily dexamethasone administration (Day 0), on the day of infection (Day 20), after randomization of mice into treatment groups (Day 50) and when mice were euthanized (Day 65). The results shown are only from uninfected mice that did not receive any DC preparations. Statistical analysis at day 20, 50, and 65 was done comparing the leukocyte levels to those measured before dexamethasone treatments ^∗∗∗^*p* < 0.001. Analyses were by one-way ANOVA followed by Tukey’s post-test.

**FIGURE 5:**
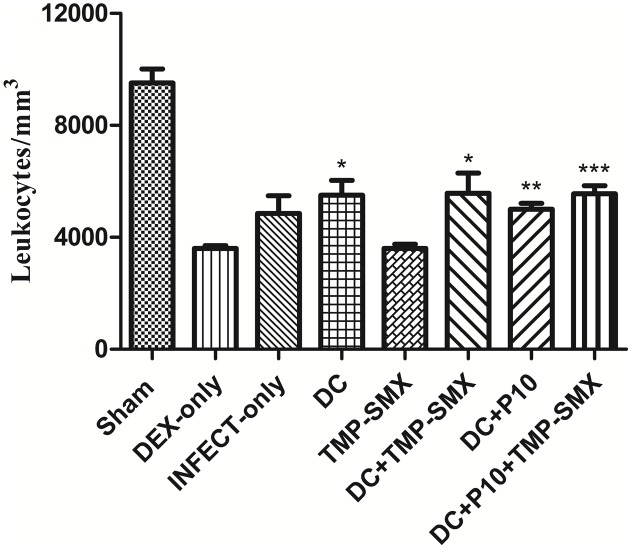
Total leukocyte counts in peripheral blood of BALB/c mice treated with dexamethasone and infected with *P. brasiliensis*. Leukocyte counts were obtained after mice were euthanized (Day 65). Except for Sham, all groups were immunosuppressed with dexamethasone (DEX). Data show Sham (uninfected without immunosuppression or any treatments), DEX-only (uninfected and untreated), INFECT-only (infected and untreated), DC (infected and treated with non-pulsed DCs), TMP-SMX (infected and treated with sulfamethoxazole-trimethoprim), DC + TMP-SMX (infected and treated with DCs and trimethoprim-sulfamethoxazole), DC + P10 (infected and treated with P10-pulsed DCs) and DC + P10 + TMP-SMX (infected and treated with P10-pulsed DCs with trimethoprim-sulfamethoxazole). Data shown are representative of two independent experiments, analyzed by one-way ANOVA followed by Tukey’s post-test, where ^∗^*p* < 0.05, ^∗∗^*p* < 0.01 or ^∗∗∗^*p* < 0.001 in comparison to DEX-only.

Dexamethasone administered in water *“ad libitum”* to mice over a 65-day period significantly reduced the peripheral leukocyte counts in a sustained manner (**Figure 4**). **Figure 5** depicts leukocyte counts on day 65, demonstrating decreased leukocyte counts in all groups in comparison with non-immunosuppressed mice (Sham). Leukocyte levels of immunosuppressed animals (DEX-only) were statistically different from all groups treated with DCs (DC, DC + TMP-SMX, DC-P10, DC + P10 + TMP-SMX). All of the DC administration conditions increased leukocyte levels in comparison to dexamethasone treated mice (DEX only). However, treatment with DCs did not significantly increase leukocyte levels in comparison to INFECT-only group.

### Immunization with P10-Primed DCs Reduces Fungal Burden

**FIGURE 6:**
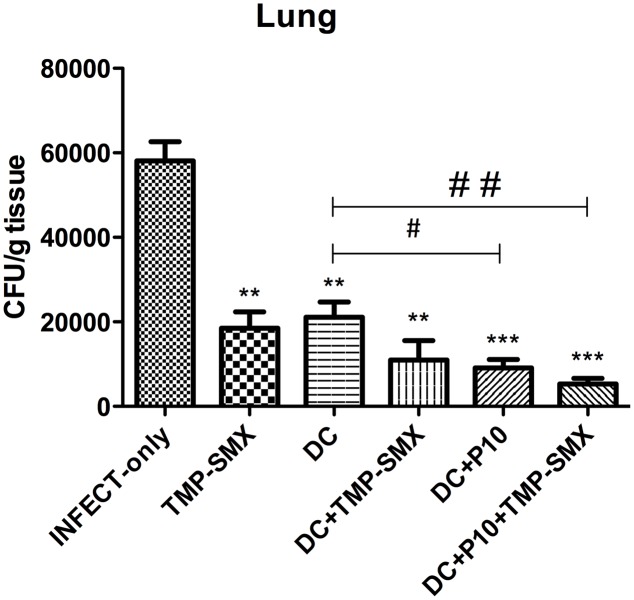
Therapeutic delivery of P10-primed DCs significantly reduces pulmonary fungal burden. Immunosuppressed mice (*n* = 6) were randomized to different treatment groups 30 days after intratracheal infection with *P. brasiliensis.* The treatment groups were INFECT-only (infected and untreated), TMP-SMX (treated with sulfamethoxazole-trimethoprim); DC (treated with non-pulsed dendritic cells); DC + TMP-SMX (treated with non-pulsed DCs and TMP-SMX); DC + P10 (treated with P10-pulsed DCs); DC + P10 + TMP-SMX (treated with P10-pulsed DCs and TMP-SMX treated). Data shown are representative of two independent experiments, analyzed by one-way ANOVA followed by Tukey’s post-test, where ^∗∗^*p* < 0.01 or ^∗∗∗^*p* < 0.001 in relation to INFECT-only and ^#^*p* < 0.05 or ^##^*p* < 0.01 in relation to DC.

The pulmonary fungal burden of immunosuppressed mice infected with *P. brasiliensis* was significantly reduced by administration of either TMP-SMX or unprimed DCs (**Figure 6**). The combination of unprimed DCs and TMP-SMX further reduced CFUs. However, the greatest reduction occurred when DCs primed with P10 were administered and this effect was further augmented when TMP-SMX was added to therapy.

### Pattern of Cytokines Induced by *In Vivo* Treatment Using Dendritic Cells Pulsed with P10

**FIGURE 7:**
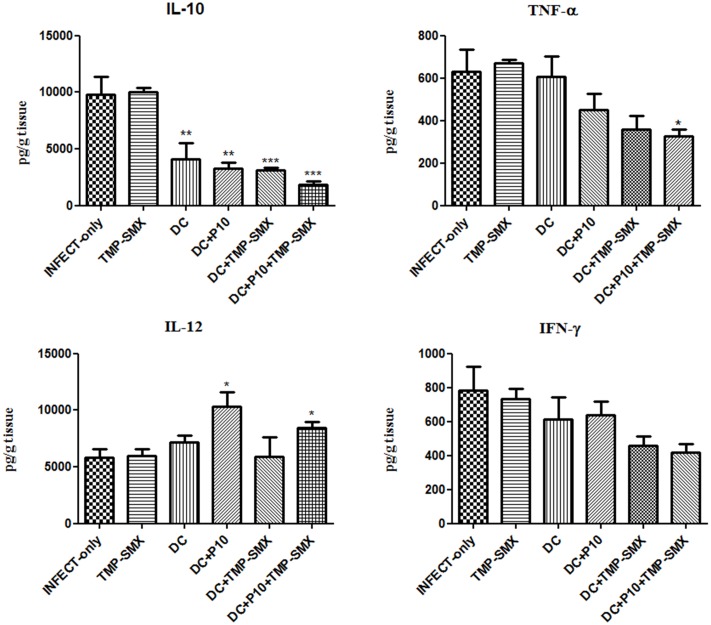
Therapeutic administration of P10-primed DCs alters cytokine responses in the lungs of immunosuppressed mice infected with *P. brasiliensis*. IL-10, TNF-α, IL-12, and IFN-γ cytokines were assayed in the lungs of mice 45 days after intratracheal infection. Immunosuppressed animals (*n* = 6) were randomized into different groups: INFECT-only (infected and untreated), TMP-SMX (treated with sulfamethoxazole-trimethoprim); DC (treated with non-pulsed dendritic cells); DC + TMP-SMX (treated with non-pulsed DCs and TMP-SMX); DC + P10 (treated with P10-pulsed DCs); DC + P10 + TMP-SMX (treated with P10-pulsed DCs and TMP-SMX treated). Data shown are representative of two independent experiments, analyzed by one-way ANOVA followed by Tukey’s post-test, where ^∗^*p* < 0.05, ^∗∗^*p* < 0.01, or ^∗∗∗^*p* < 0.001 in comparison to INFECT-only.

IL-10, IL-12, IFN-γ, and TNF-α levels were measured by ELISA in the lungs of mice with different treatment regimens (**Figure 7**). A significant reduction of IL-10 was observed in all groups that received DCs with the greatest reduction occurring in mice that received P10-primed DCs together with TMP-SMX. Increased levels of IL-12 occurred in mice treated with P10-primed DCs with or without TMP-SMX treatment. Levels of TNF-α were reduced after the administration of DCs with TMP-SMX or primed DCs, but were only significantly reduced in the combination of primed DCs with TMP-SMX treatment. No significant differences were detected in IFN-γ levels, although there was a trend to lower levels in the groups treated with TMP-SMX combined with either P10-primed or unprimed DCs.

### Analysis of Histological Sections

**FIGURE 8:**
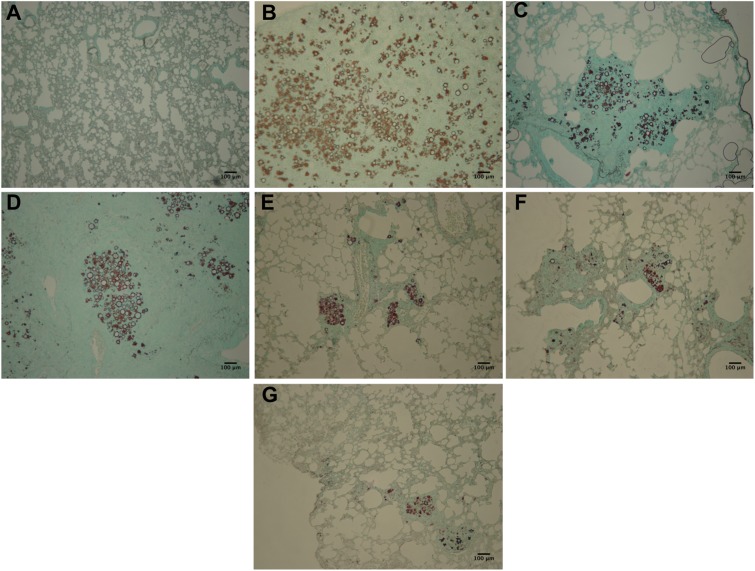
Gomori-Grocott staining reveals that therapy with P10-primed DCs reduces pulmonary fungal load in *P. brasiliensis* infected mice. Representative Gomori-Grocott stained lung sections from immunosuppressed mice 45 days after intratracheal infection with *P. brasiliensis*. **(A)** uninfected and untreated animals; **(B)** infected and untreated; **(C)** infected and treated with TMP-SMX; **(D)** infected and treated with non-pulsed DCs; **(E)** infected and treated with P10-pulsed DCs; **(F)** infected and treated with non-pulsed DCs in combination with TMP-SMX; **(G)** infected and treated with P10-pulsed DCs in combination with TMP-SMX. Photographs were taken at 300× magnification. Bars denote 100 μm.

Lung sections were subjected to Gomori-Grocott and HE staining. Gomori-Grocott stained micrographics (**Figure 8**) depict the fungal burden, showing the presence of fungal cells in lung tissue. **Figure 8A** demonstrates uninfected immunosuppressed mice with an absence of fungal cells. Abundant fungal cells were present in tissues of immunosuppressed mice in the following groups: infected and not treated (**Figure 8B**), infected and treated with (**Figure 8C**) and non-pulsed DCs treated groups (**Figure 8D**). In contrast, lung sections from immunosuppressed and infected animals treated with P10-pulsed DCs (**Figure 8E**), non-pulsed DCs and TMP-SMX (**Figure 8F**) and P10-pulsed DCs and TMP-SMX (**Figure 8G**) had a significantly lower fungal burden. Moreover, the animals that received P10-pulsed DCs (**Figures 8E,G**) presented the lowest burden of fungal cells in lung tissue.

**FIGURE 9:**
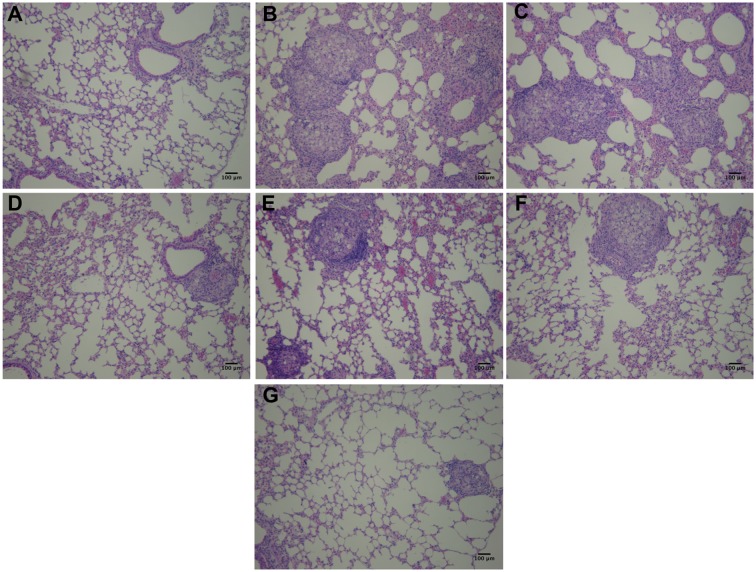
Hematoxylin and Eosin staining reveals that therapy with P10-primed DCs reduces pulmonary damage in mice infected with *P. brasiliensis*. Representative Hematoxylin/Eosin stained lung sections from immunosuppressed mice 45 days after intratracheal infection with *P. brasiliensis*. **(A)** uninfected and untreated animals; **(B)** infected and untreated; **(C)** infected and treated with TMP-SMX; **(D)** infected and treated with non-pulsed DCs; **(E)** infected and treated with P10-pulsed DCs; **(F)** infected and treated with non-pulsed DCs in combination with TMP-SMX; **(G)** infected and treated with P10-pulsed DCs in combination with TMP-SMX. Photographs were taken at 300× magnification. Bars denote 100 μm.

Hematoxylin/eosin staining (**Figure 9**) revealed normal tissue architecture in non-infected mice (**Figure 9A**). It was observed the presence of granulomas with fungal cells and intense inflammatory infiltrates in lung parenchyma of immunosuppressed and infected-only (**Figure 9B**), infected and treated with TMP-SMX (**Figure 9C**) and infected and treated with P10-pulsed DCs (**Figure 9E**) groups. Granuloma-like structures were also observed in the other infected groups, but inflammatory infiltrates were reduced in the non-pulsed DCs (**Figure 9D**), non-pulsed DCs and TMP-SMX (**Figure 9F**) and P10-pulsed DCs and TMP-SMX treated (**Figure 9G**) groups. Lung parenchyma was significantly best preserved and less compromised in P10-pulsed DCs and TMP-SMX treated (**Figure 9G**) group.

## Discussion

Acute and sub-acute forms of PCM are highly aggressive in the absence of early antifungal treatment immediately after the disease manifestation and death remains a relatively common outcome in this setting. Antifungal therapy requires a long and exhausting approach to prevent relapses. In addition, standard therapeutic regimens are associated with significant toxicities, such as nephrotoxicity and hepatotoxicity, and intense therapy does not prevent the development of disease sequelae, such as pulmonary fibrosis or scarring (Shikanai-Yasuda et al., 2006; Bocca et al., 2013; Buccheri et al., 2015). Hence, antifungal therapy alone does not inhibit the intense inflammatory responses in infected tissues that occur in response to the release of fungal antigens from dead or dying cells resulting in extensive tissue damage, which, in fact, can lead to death (Benard et al., 2012). To combat this adverse host response, we have been studying a therapeutic vaccine against *P. brasiliensis* based on a peptide known as P10, which induces a strong, protective immunological response that significantly reduces fungal burden, tissue injury and fibrosis in a murine experimental PCM model (Travassos et al., 2008a; Taborda et al., 2015).

Dendritic cells are potent stimulators of the immune response and are able to activate naïve T cells by presenting peptide antigens (Banchereau, 2000; Thind et al., 2015). Previously, we have demonstrated that intravenous or subcutaneous injection of P10-primed DCs into mice with established PCM led to a significant reduction in fungal burden and stimulated a Th_1_-biased cytokine response (Magalhães et al., 2012). Here we demonstrated that *in vitro* DC stimulation with P10 increases the frequency of CD80^+^/CD86^+^ DCs, while also increasing the production of IL-12 by these cells. These data support our hypothesis that P10 stimulation activates DCs resulting in improved antigen presentation and T-cell responses that culminate in protection against *P. brasiliensis* infection.

In the present study, we also evaluated the therapeutic potential of P10-primed DCs in the setting of acute/subacute PCM in immunocompromised mice using dexamethasone, which effectively reduces airway inflammation through multiple mechanisms (Muñoz et al., 2014). Dexamethasone administered in drinking water for 20 days reduced the total number of leukocytes by ∼40%. Infecting these mice with *P. brasiliensis* Pb18 induced an aggressive disease that was similar to the acute/subacute form of PCM. In fact, dexamethasone immunosuppressed mice infected with 3 × 10^5^ yeast cells of Pb18 have 100% mortality within 80 days (Muñoz et al., 2014). The administration of P10-primed DCs to dexamethasone treated, Pb18 infected mice significantly reduced the fungal burden, with the greatest reduction being achieved with or without concomitant antifungal treatment. These results were supported by the histological evaluations that revealed the dramatic protection of the lung architecture along with significant reductions in yeast cell numbers in mice treated with primed DCs, especially in the setting of antifungal combination therapy.

Protection against systemic fungal infection requires an effective cell immune response and the presence of IL-12 and IFN-γ appear to be essential to control the progression of PCM (Travassos and Taborda, 2012a). We have previously demonstrated that immunization with P10 can restore lymphoproliferation in immunosuppressed animals (Muñoz et al., 2014). In these prior experiments, murine DCs pulsed *in vitro* with P10 or IFN-γ (used as a control) induced significant IL-10, IL-12 production and enhanced MHC-II expression. In general, DCs stimulated with IFN-γ induces mature cells to preferentially produce IL-12, whereas DCs stimulated by IL-10 and prostaglandin E2 generate low amounts of IL-12 (Banchereau, 2000). In our present study, lung homogenates from immunosuppressed and Pb18 infected mice treated with DCs pulsed with P10, with or without antifungal drugs, contained significantly higher levels of IL-12 and significant reduction of IL-10 levels compared with the lungs of immunosuppressed and infected mice that did not receive DCs. The therapeutic use of P10-primed DCs in our non-immunosuppressed murine PCM model also led to increased levels of IL-12 and reduced IL-10 levels in lung tissues, albeit high levels of IFN-γ were also present at the time the mice were euthanized in the prophylactic an therapeutic model (Magalhães et al., 2012). Similarly, we have found significant increased levels of IFN-γ and IL-12 and reduction of IL-10 level in the lungs of mice vaccinated with 20 μg of P10 in complete Freund’s adjuvant that were subsequently infected with Pb18 (Muñoz et al., 2014). We believe that the peak of IFN-γ expression is likely occurring at different times in these different models, in which mice were also euthanized at different time points after infection. This hypothesis is supported by the present results, in which we found significant reductions of fungal cells when P10-pulsed DCs were administered with or without concomitant antifungal therapy.

## Conclusion

Out data indicate that P10-pulsed DCs, in the presence or not of antifungal drug, have the ability to induce specific immunity against *P. brasiliensis* in animals previously treated with corticosteroid in an experimental model that mimics the most aggressive form of PCM. These findings further support the active pursuit of P10 as a candidate for a human vaccine.

## Author Contributions

LS performed experiments, analyzed data, and wrote the manuscript. AS, LD, GR, and JM performed experiments. JN, LT, and CT revised the manuscript. CT designed the experiments, wrote and revised the manuscript.

## Conflict of Interest Statement

The authors declare that the research was conducted in the absence of any commercial or financial relationships that could be construed as a potential conflict of interest.
